# Collagen-Fibrinogen Lyophilised Scaffolds for Soft Tissue Regeneration

**DOI:** 10.3390/ma10060568

**Published:** 2017-05-23

**Authors:** Jennifer Shepherd, Daniel Bax, Serena Best, Ruth Cameron

**Affiliations:** Department of Materials Science and Metallurgy, University of Cambridge, Cambridge CB3 0FS, UK; dvb24@cam.ac.uk (D.B.); smb51@cam.ac.uk (S.B.); rec11@cam.ac.uk (R.C.)

**Keywords:** collagen, fibrinogen, adhesion, lyophilisation, micro-computed tomography

## Abstract

A significant body of research has considered collagen as a scaffold material for soft tissue regeneration. The main structural component of extra-cellular matrix (ECM), collagen’s advantages over synthetic polymers are numerous. However, for applications where higher stiffness and stability are required, significant cross-linking may affect bioactivity. A carbodiimide (EDC) cross-linking route consumes carboxylate groups that are key to collagen’s essential cell recognition motifs (GxOGER). Fibrinogen was considered as a promising additive as it plays a key role in the process of wound repair and contains RGD integrin binding sites which bind to a variety of cells, growth factors and cytokines. Fibrinogen’s binding sites however, also contain the same carboxylate groups as collagen. We have successfully produced highly interconnected, porous collagen-fibrinogen scaffolds using a lyophilisation technique and micro-computed tomography demonstrated minimal influence of either fibrinogen content or cross-linking concentration on the scaffold structure. The specific biological effect of fibrinogen additions into cross-linked collagen are considered by using films as a model for the struts of bulk scaffolds. By considering various additions of fibrinogen to the collagen film with increasing degrees of cross-linking, this study demonstrates a significant biological advantage with fibrinogen addition across the cross-linking concentrations typically applied to collagen-based scaffolds.

## 1. Introduction

A key desire in tissue engineering is to mimic and regenerate the existing native environment. The extra-cellular matrix provides physical support to tissues by occupying the intercellular space and acting as a native scaffolding, but is also a highly dynamic, mobile, and flexible structure which is key to cellular behaviour and tissue function [1]. Collagen forms the major structural component of this and, as such, there has been a significant focus of research on collagen as a scaffold material for tissue regeneration [2,3,4,5]. However, in order to more closely replicate the ECM chemistry of specific tissues, there has been widespread consideration of the addition of other biologically-relevant materials. Glycosaminoglycans (GAGs), such as chondroitin-6-sulphate or hyaluronic acid, for example, have been considered extensively for skin [6,7,8,9,10] and musculoskeletal engineering applications [11,12,13], with hyaluronic acid additionally being considered for adipose tissue engineering [14]. GAGs form porous hydrated gels and tend to fill most of the extra-cellular matrix space providing mechanical support to tissues whilst also allowing for the migration of cells and diffusion of water soluble molecules [7]. GAGs may also provide mechanical advantage and improved stability [15]. Elastin, with its highly elastic nature, is found naturally in cardiovascular tissue and has also received some attention [16,17]. 

Fibrinogen is a soluble 340 kDa protein found in the blood and plays a key role in blood clotting and platelet aggregation [18]. Following vascular injury, fibrinogen is cleaved by thrombin to form fibrin, the most abundant protein of blood clots. Various cleavage products of fibrinogen have been found to be released during coagulation and fibrinolysis [18], meaning it is often considered as a haemostatic agent or for wound dressing [19,20,21]. Fibrinogen is a protein with the capacity to bind to a wide array of proteins and it has been shown to regulate cell adhesion and spreading and display vasoconstrictor and chemotactic activities [18]; its application in tissue engineered scaffolds could, therefore, be beneficial particularly for vascular prosthetic engineering [22]. Whilst there are perhaps some concerns with the immunological response of fibrinogen within the central nervous system, and even links with multiple sclerosis, soluble fibrinogen within the bloodstream has not been observed to be proinflammatory [23]. As summarised by Sell et al., fibrinogen contains RGD binding sites which commonly bind fibroblasts and endothelial cells, and it also binds with high affinity to VEGF (vascular endothelial growth factor), FGF (fibroblast growth factor), and a number of other cytokines [22]. 

A number of studies on electrospun fibrinogen scaffolds have demonstrated high levels of bioactivity with ready migration, deposition of native collagen, and rapid remodelling [24,25,26,27], but production of fibrinogen scaffolds through other routes is problematic [28]. Fibrin gels have received some attention [29,30] but the lack of structural integrity limits practical use [31]. Additionally degradation rates are rapid, with some fibrin gels dissolved within just days [32]. It, thus, appears logical to consider fibrinogen as a secondary addition to more structurally-relevant biopolymers, such as collagen. Whilst gelatin-fibrinogen cryogels have been considered for wound repair [33], a fibrinogen-coated collagen patch for treatment of thoracic aortic aneurysm [34] and a number of collagen-fibrin composites designed [35,36] the application of fibrinogen as a secondary addition in combination with collagen warrants far greater consideration.

When collagen is generated in vivo, covalent inter- and intra-molecular bonds are formed as a result of enzymatic and non-enzymatic cross-linking [37]. These cross-links are essential for mechanical strength and proteolytic resistance and are not formed during the self-assembly or reconstitution of collagen in neutral pH. The zero length cross-linker 1-ethyl-3-(3-dimethylaminopropyl) carbodiimide (EDC) in combination with *N*-hydroxysuccinimide (NHS) has been shown to be a highly effective route for the cross-linking of collagen structures [9,10,38]. Whilst a minimal effect of bioactivity is generally reported with the EDC cross-linking of collagen structures [38,39], higher concentrations of the cross-linking agents have been associated with cytotoxicity [7,10]. In a recent study on extruded collagen fibres cross-linked with the EDC-NHS chemistry, a significant increase in the attachment of human tenocytes and a more flattened phenotypical appearance was associated with a reduction in the cross-linking concentration [40]. 

In 1996 Olde-Daminck et al. investigated the optimum concentrations of reagents necessary for the carbodiimide-based cross-linking of ovine-derived dermal collagen [41]. They determined that a ratio of 5:2:1 EDC:NHS:carboxylic acid groups in collagen resulted in the highest degree of cross-linking and this has become something of a standard when it comes to cross-linking concentrations for collagen scaffolds, certainly within our research group [14,42,43,44,45]. Recent work, however, has suggested that a lower concentration provides a better biological response with acceptable mechanics and stability [46,47].

As Davidenko et al. describe, there is a tendency for studies of cell attachment to protein-derived matrices, such as collagen, to ignore the detailed mechanism of binding. There is very rarely any consideration of whether binding is non-specific or integrin-mediated and, if the latter, which integrins are responsible [47]. This can become particularly relevant when cross-linking is considered. In EDC cross-linking, carboxylate groups on the amino acid side chains of glutamate (E) or aspartate (D) are consumed. These groups form an integral part of the cell-recognition motifs of both collagen and fibrinogen; collagen includes the triple-helical GxOGER sequence (where G is glycine; O is hydroxyproline; R is arginine and **x** is hydrophobic, exemplified by phenylalanine, F) and fibrinogen contains a linear RGD motif. In the recent work of Davidenko et al., carbodiimide cross-linking was found to ablate integrin-dependent cell activity on both two-dimensional and three-dimensional architectures of collagen and gelatin [47]. Gelatin, like fibrinogen, binds using a linear RGD cell adhesive motif and, thus, it is reasonable to assume that EDC cross-linking will affect the activity of the fibrinogen in addition to that of the collagen.

Collagen-fibrinogen based scaffolds would appear to offer considerable biological advantage not least in terms of the multiple available cell-recognition motifs. This work, therefore, considers the feasibility of applying lyophilisation to the production of collagen-fibrinogen scaffolds and carries out structural analysis investigating the effect of both fibrinogen content and cross-linking concentration on pore structure. Whilst previous work by Ber et al. has considered the adsorption of fibrinogen to the surface of cross-linked collagen films in a secondary process [48], in this study we aim to produce scaffolds with an intimate mixture of collagen and fibrinogen in a single step lyophilisation process, thus adding no additional complexity to production. Additionally, this work investigates the specific biological effect of fibrinogen additions into cross-linked collagen films. These films can be treated as a model for the struts of bulk scaffolds [49,50] providing information at the cellular length scale highly relevant to cell behaviour on scaffolds and providing a simpler system where the effect of chemistry can be more straightforwardly elucidated. Whilst common cell types typically exhibit fairly broad reactivity to a range of integrins [51,52], this study concentrates on a C2C12 mouse myoblast cell line with specific RGD binding receptors, thus, they should adhere specifically to fibrinogen. These cells have, in a previous study, been shown to exhibit minimal adhesion to collagen films of any cross-linking concentration [47]. By considering various additions of fibrinogen to the collagen film with increasing degrees of cross-linking, this study aims to investigate whether fibrinogen can impart any biological advantage across the cross-linking concentrations typically applied for collagen-based scaffolds.

## 2. Results

### 2.1. Production of Collagen–Fibrinogen Scaffolds and Films

**Figure 1:**
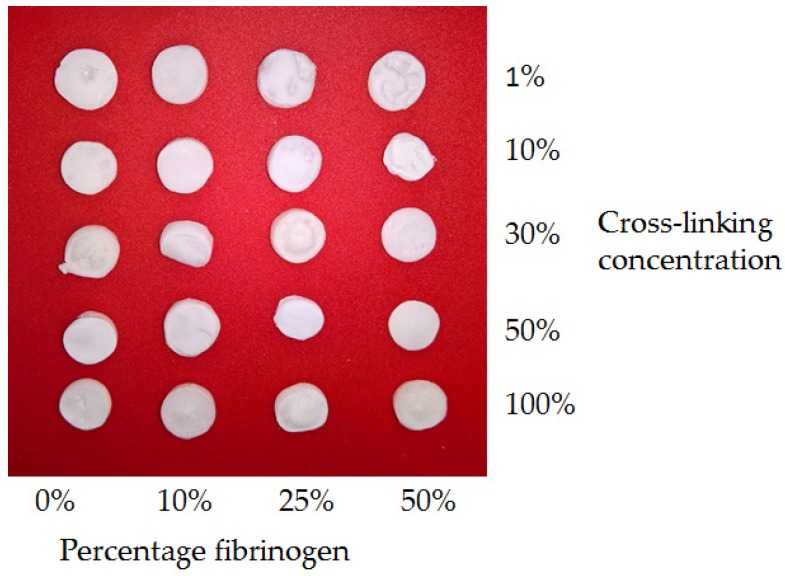
Photographs demonstrating typical samples of the full range of freeze-dried scaffolds. Percentage cross-linking concentrations refer to proportion of standard cross-linking concentration as defined above. Gross structures were comparable and the variation in shrinkage associated with freeze-drying was insignificant.

Lyophilised collagen-fibrinogen scaffolds were successfully produced with between 0% and 50% fibrinogen (0, 10, 25, and 50 wt %). and cross-linking carried out using the EDC/NHS chemistry. 100% cross-linking concentration referred to the standard ratio of 5:2:1 EDC:NHS:carboxylic acid groups in the collagen and fibrinogen. Reduced cross-linking concentrations of 1%, 10%, 30%, and 50% were also analysed. Scaffolds showed comparable gross structures (Figure 1) and variation in shrinkage associated with the freeze-drying process was not significant. Films with these compositions and 30% cross-linking concentration were additionally produced on glass coverslips. 

**Figure 2:**
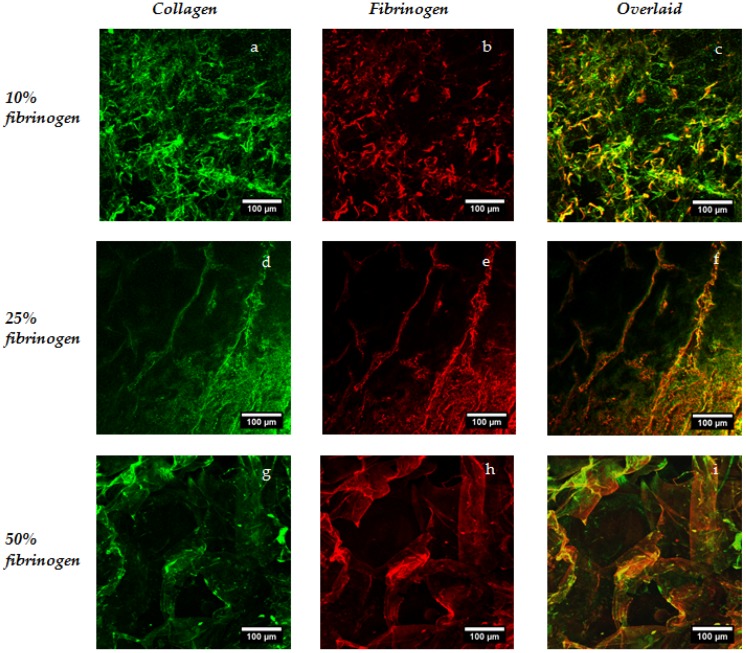
Investigation of fibrinogen distribution in scaffolds using immunofluorescence. These representative confocal images are taken after incubation with primary antibodies for both collagen and fibrinogen and associated secondary antibodies.

**Figure 3:**
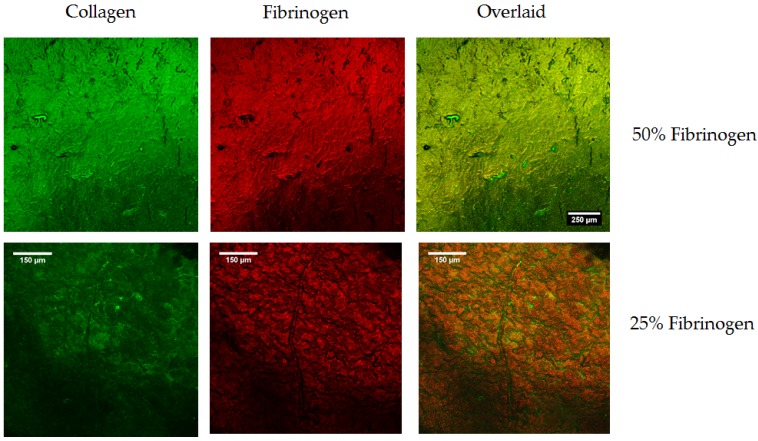
Investigation of fibrinogen distribution in films using immunofluorescence. Again, representative images of the films are taken after incubation with primary antibodies for both collagen and fibrinogen and associated secondary antibodies.

In order to investigate spatial distribution of the two constituents within scaffolds and films, a simultaneous immunofluorescence procedure was carried out and samples were imaged in the hydrated state using confocal microscopy. The collagen associated secondary carried an Alexa Fluor 488 fluorophore, and the fibrinogen carried an Alexa Fluor 648 fluorophore (all antibodies from Abcam, Cambridge, UK), hence, the collagen is shown in green in the images of Figure 2 and Figure 3 and fibrinogen in red; the separate channels along with the overlaid images are included in the figures. The chemical distribution and the success of the immunofluorescence procedure was found to be independent of the cross-linking concentration. In these images of 10%, 25%, and 50% fibrinogen scaffolds, a comparatively uniform distribution of the two constituents was observed throughout the scaffold structure, with dark areas (low signal intensity) being the result of open pores. Imaging of the composite films can be treated as the analogue of the scaffold struts and the simpler structures perhaps provide a better picture of the good level of mixing of the constituent reagents and, consequently, a consistent chemical environment for cellular analysis. 

#### 2.1.1. Structural Analysis with Micro-CT and Scanning Electron Microscopy (SEM) 

**Figure 4:**
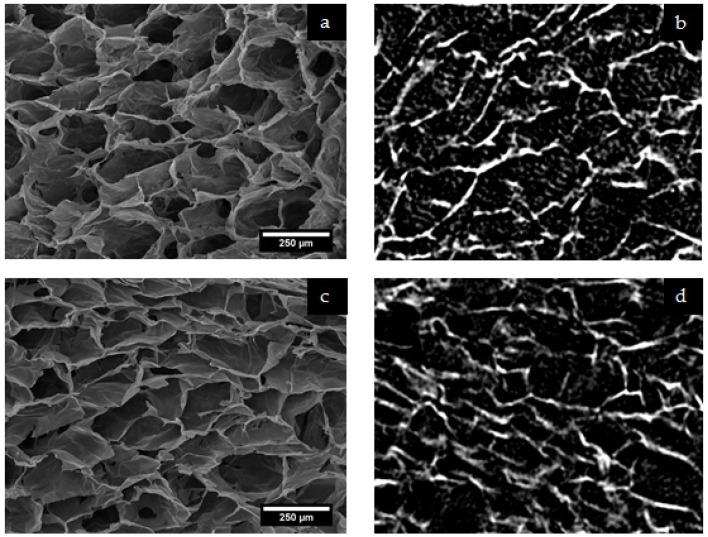
Characteristic pore structures for lyophilized collagen-fibrinogen scaffolds as imaged directly using SEM ((**a**) and (**c**)) and generated from the reconstructed micro-CT data ((**b**) and (**d**)). Micro-CT images are at the same magnification as those from SEM. Images (**a**) and (**b**) are of a scaffold with 10% cross-linking and 10% fibrinogen, and images (**c**) and (**d**) of a scaffold with 100% cross-linking and 50% fibrinogen.

Scaffolds with the full combination of compositions and cross-linking concentrations were scanned without sectioning using a Skyscan 1172 system (Bruker Micro-CT, Kontich, Belgium). After reconstruction, scaffold structures were observed and 1 mm^3^ cubic regions of interest were selected for porosity analysis using CTan (Bruker Micro-CT, Kontich, Belgium). Porosity analysis was carried out in order to determine whether any structural variation was associated with changing fibrinogen content or cross-linking concentration. Imaging of sections of the scaffolds were also carried out using SEM. Figure 4 includes representative SEM images and single slices from the reconstructed micro-CT data and demonstrates good correlation between the structures as analysed using the two techniques. 

The mean pore size was found to vary significantly within the samples considered, with mean values of between ca. 60 and 140 microns calculated; however, this was not associated with a systematic dependence upon either fibrinogen content or cross-linking concentration. Perhaps of greater interest, in terms of cell migration, is the notion of percolation diameter. The percolation diameter is the diameter of the largest sphere able to penetrate through an infinitely large scaffold and is a scalable measure of pore interconnectivity [42]. Mean values for the percolation diameter varied between 80 and 120 μm, but with significant associated standard deviations, no significant effect of either fibrinogen content or cross-linking concentration were observed. It, thus, appears reasonable not to apply too much weight to the consideration of variations in pore structure during future analysis of the biological response of these materials.

### 2.2. Films for Cellular Analysis

Collagen-fibrinogen films with all fibrinogen concentrations and cross-linking at the 10% and 100% level were successfully produced on the base of 48-well plates for analysis of cellular adhesion and spreading. These films were designed as a model of the strut walls of the three-dimensional structures and a first step in biological analysis. Cross-linking concentrations were selected so as to consider the biological effects of the fibrinogen addition at low and high cross-linking concentrations and, thus, infer something of the biological effect of fibrinogen additions across relevant cross-linking compositions.

#### 2.2.1. Cellular Adhesion and Spreading

**Figure 5:**
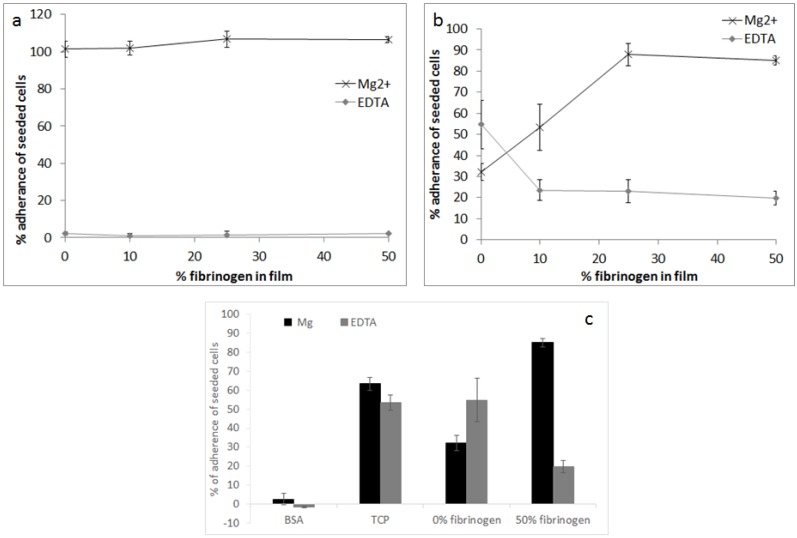
Percent adherence of seeded cells as a function of the percent of fibrinogen in film (**a**) at 10% cross-linking concentration; and (**b**) 100% cross-linking concentration; and (**c**) a comparison of the percent adhesion to bovine serum albumin (BSA) and tissue cultured plastic (TCP) controls at 100% cross-linking concentration. Adhesion is shown in the presence of 5 μM Mg^2+^ or EDTA.

Cellular adhesion was investigated using a C2C12 mouse myoblast cell line with specific RGD binding receptors and in the presence of either Mg^2+^ or EDTA in order to isolate integrin (Mg^2+^) versus non-integrin (EDTA) adhesion. The effect of fibrinogen content was considered at 10% (Figure 5a) and 100% cross-linking concentrations (Figure 5b).

In the case of the 10% cross-linking concentration in the presence of Mg^2+^, cells were observed to be highly adherent even in the absence of fibrinogen. Non-specific adhesion (in the presence of EDTA) was low in the case of low cross-linking concentration. In the case of the 100% cross-linking concentration, the percentage of adherent cells increased linearly between 0% and 25% fibrinogen, before plateauing with around 90% adherent cells at 50% fibrinogen. This specific adhesion at elevated fibrinogen levels was higher even than the positive control TCP (tissue cultured plastic) (Figure 5c). Non-specific adhesion (in the presence of EDTA) was significantly increased with the higher cross-linking concentration and decreased with increasing fibrinogen content. 

**Figure 6:**
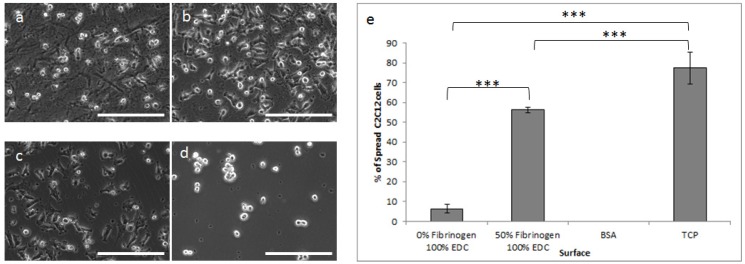
Phase contrast images demonstrating cell spreading on (**a**) 0% fibrinogen; (**b**) 50% fibrinogen and controls; (**c**) tissue cultured plastic and (**d**) bovine serum albumin. (**e**) Percentage of spread cells on the various surfaces (***p < 0.0001). All films were cross-linked at 100%. Scale bar = 200 µm.

As a consequence of the very clear effect of fibrinogen concentration on cell adhesion at the maximum cross-linking level, cell spreading was investigated using only this cross-linking concentration. In a quantitative analysis 0% and 50% fibrinogen-containing films were compared to negative control bovine serum albumin (BSA) and positive control tissue cultured plastic (TCP). All cell spreading was investigated in the presence of Mg^2+^ to consider only specific binding. With phase contrast, cells on the 0% fibrinogen surface were observed to be highly rounded (Figure 6a) with an approximately 10-fold increase in cell spreading observed on the films containing 50% fibrinogen (Figure 6b,e) compared to the collagen-only material. 

**Figure 7:**
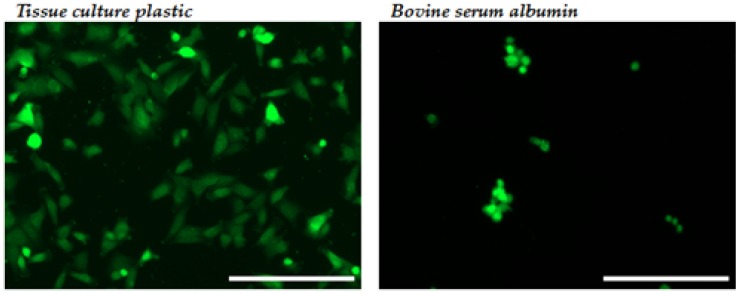

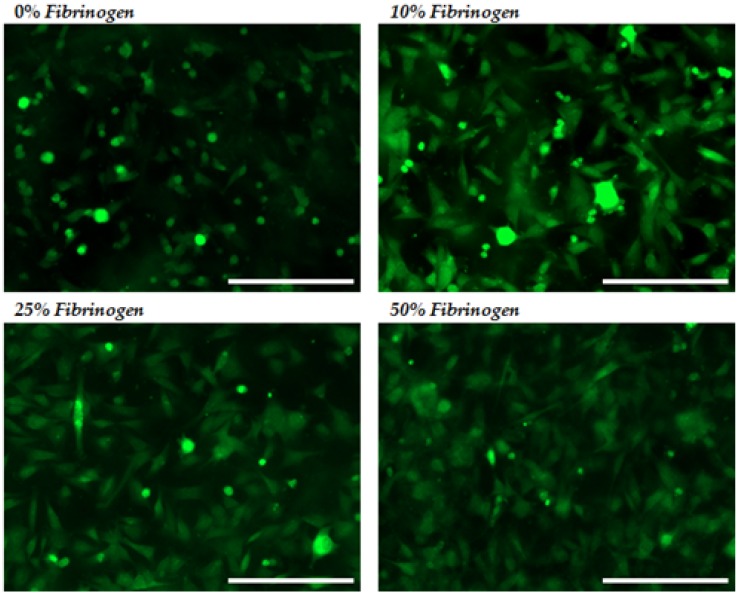
Spreading of CellTracker-stained C2C12 cells on 100% cross-linked films with increasing fibrinogen concentration compared to the negative control (BSA) and positive control (tissue-culture plastic). Scale bar = 200 μm.

The nature of the cell spreading was further investigated across the fibrinogen additions by way of CellTracker Green CMFDA prestaining of the cells (Figure 7). The number of rounded cells was observed to decrease significantly with increasing fibrinogen concentration with highly-spread cells observed at the 25% and 50% fibrinogen levels.

## 3. Discussion

A significant focus of biomaterials research is in the development of more physiologically-relevant templates for tissue regeneration, whether this is in terms of chemistry, structure, or mechanics. Collagen, as the major structural component of soft tissues, remains an obvious choice for scaffold material and fibrinogen with its significant role in wound repair and favourable adhesion to a range of cells and growth factors appeared a logical secondary addition. In this work we have not only demonstrated the production of novel lyophilized collagen-fibrinogen scaffolds across a range of compositions and EDC-NHS cross-linking chemistries, but have also carried out characterization in a targeted fashion, whether in terms of the spatial distribution of the constituents, structural analysis, or cellular response. 

Of the many papers to have considered the addition of GAGs or other biologically-relevant materials to collagen-based scaffolds, the spatial distribution of the constituent components is considered only very rarely [53]. This distribution is clearly key not only to biological response, but also in terms of mechanical and structural homogeneity. Ma et al. applied fluorescent staining in the investigation of collagen and chitosan distribution within scaffolds for skin tissue engineering [52]. Rhodamine-labelled collagen and FITC-labelled chitosan were prepared by mixing the fluorescent additives into the biomacromolecule solutions prior to lyophilisation. This method required a four-week period where free dyes were dialysed off in 0.05 M acetic acid solution. Samples were imaged using confocal microscopy and a highly uniform distribution of the two components observed. Immunofluorescence is a standard technique widely applied across microbiology, cell biology, and tissue engineering whereby highly specific antibodies adhere only to the molecules of interest and fluorescent markers excite at specific wavelengths for confocal or fluorescent imaging. As both collagen and fibrinogen are extremely common biomolecules, those specific antibodies are well developed and the novel application to engineered scaffolds provided a comparatively simple and highly-accurate method of the determination of chemical special distribution.

Analysis of the biological response of a scaffold material is a complex mix of chemistry, pore structure, as well as strut surface topography [54,55]. Literature has demonstrated that scaffold structure has a significant effect on cellular response hence the analysis of pore structure variation carried out here. Murphy et al. investigated the effect of mean pore size in collagen-GAG lyophilised scaffolds on osteoblast adhesion and early stage migration. While an early peak in cell adhesion was observed at the smaller pore sizes (120 microns), perhaps as a result of higher specific surface area, larger pore sizes (300 micron+) were generally associated with improved cell migration [56]. The structure is important in terms of both nutrient transfer and development of a vascular network and for cellular migration. In a study by Karageorgiou and Kaplan pore size was shown to affect the progression of osteogenesis. Small pores favoured hypoxic conditions and induced osteochondral formation before osteogenesis, while large pores, that were well-vascularized, led to direct osteogenesis (without preceding cartilage formation) [57]. Micro-computed tomography was, thus, carried out in our current study in order to investigate whether either fibrinogen content or cross-linking concentration had any systematic and significant effect on the pore structure. Although variation in the mean pore sizes were significant, there was no correlation with either of the two considered variables.

Whilst pore size is an important variable for consideration, of greater interest perhaps, is the idea of scaffold interconnectivity. It has recently been demonstrated by Ashworth et al. that pore size and interconnectivity can be independently controlled within a freeze-dried scaffold. In a cell invasion study with primary human fibroblasts in samples with comparable pore sizes, smaller percolation diameter (reduced interconnectivity) were associated with markedly reduced cell invasion. As Ashworth et al. state, optimization of pore size should be considered ‘an incomplete approach to the enhancement and control of cell invasion, and that an understanding of interconnectivity is required to ensure efficient cell movement into a structure’ [42]. Percolation diameters calculated across the range of fibrinogen contents and cross-linking concentration were broadly similar, thus, it is reasonable that even with variations in mean pore size of all of the collagen-fibrinogen scaffolds could be treated as comparable structures from a cellular perspective. 

A mass of published data concerns the optimization of cross-linking chemistries for collagen-based scaffolds [10,37,45,52]. The EDC-NHS cross-linking method, whilst generally demonstrating favourable behaviour compared to other physical and chemical cross-linking methods [9,10,37], has more recently been found to ablate integrin-dependent cell activity on both 2D and 3D architectures [47,50]. The cell adhesion analysis carried out here is based upon the recent study of Davidenko et al., where the cell binding to two- and three-dimensional architectures consisting of collagen and gelatin were evaluated using cell types with specific collagen and gelatin binding integrins. Like fibrinogen, gelatin exhibits RGD integrin binding sites and cell adhesion was considered using the C2C12 cell line. Cell-lines with targeted collagen binding integrins were additionally considered and in both cases the effect was the same: carbodiimide cross-linking was found to ablate integrin-dependent cell activity on both 2D and 3D architectures. C2C12 cells should not have exhibited binding to collagens GxOGER sequence yet in the case of the low cross-linking concentration (Figure 6a) specific binding was high and independent of fibrinogen content. A previous four hour adhesion study showed no adhesion of C2C12 cells to a collagen film [58], but more recent unpublished work has supported adhesion in the cases such as these where films are produced from more fibrillar insoluble collagen. 

Given the targeted nature of the C2C12 cells, an increase in cell adhesion and spreading with the increase in fibrinogen content was to be expected. However the cell analysis was designed to elucidate whether the biological activity of the fibrinogen could successfully be maintained within a cross-linked collagen-fibrinogen scaffold. As previously mentioned, cellular analysis was carried out on films as analogues for the scaffold strut walls so as to deconvolute considerations of chemical and dimensional variation [47]. Future analysis will clearly have to take into consideration the additional complexity associated with the biological response to three-dimensional environments but the results summarised in Figure 5, Figure 6 and Figure 7 demonstrate maintenance of fibrinogen’s biological activity. With the addition of 25% or 50% fibrinogen, cells were observed to be highly adherent and well-spread even when employing cross-linking concentrations where specific binding has been shown to be ablated in collagen. Fibrinogen thus appears to offer biological advantage at high cross-linking concentrations perhaps considered for high stiffness or stability. The alternative binding sites of the fibrinogen, however, also offer a greater degree of flexibility in terms of varied cell adhesion and protein attachment across a wide range of cross-linking concentrations, thus, increasing the possible application of lyophilized collagen scaffolds.

## 4. Materials and Methods 

### 4.1. Study Design

Collagen-fibrinogen scaffolds and films were produced as summarised in Table 1.

### 4.2. Collagen–Fibrinogen Scaffolds

Lyophilised structures were produced with 0%, 10%, 25%, and 50% fibrinogen. Type I insoluble collagen from Bovine Dermis (Devro Medical, Moodiesburn, Scotland) was swollen for 48 h in 0.05 M acetic acid at 4 °C prior to blending (Waring commercial blender) and vacuum degassing in order to produce a 1 wt % suspension. Lyophilised fibrinogen (from human blood plasma, plasminogen depleted (Enzyme Research, Swansea, UK)) was dissolved in phosphate-buffered saline solution (PBS) at 37 °C to form a 1 wt % solution. The two components could be combined only through gentle mixing as anything more vigorous resulted in foaming of the fibrinogen solution. Samples were produced in standard 24-well plates (Corning, NY, USA) with 2 mL of slurry pipetted per sample.

Freeze-drying was carried out using a VirTis AdVantage Freeze dryer (SP Scientific, Warminster, PA, USA) with a cooling rate of 0.17 °C/min to a primary freezing temperature of −20 °C, and drying at 0 °C for 1200 min at a pressure of 80 mTorr. After cross-linking then rigorous washing, samples were freeze-dried, again applying the same thermal profile.

### 4.3. Cross-Linking

Cross-linking was carried out using an EDC/NHS (1-ethyl-3-(3–dimethylaminopropyl) carbo-diimide/*N*-hydroxysuccinimide) chemistry (both reagents from Fisher Scientific). 100% concentration referred to a ratio of 5:2:1 EDC:NHS:COOH groups in collagen/fibrinogen [59] with concentrations of 1%, 10%, 30%, and 50% also considered. It was felt to be important that with up to 50% fibrinogen being added to the structure, the chemistry of the fibrinogen should be taken into account when it came to the calculation of cross-linking concentrations. The fibrinogen structure is more complicated than collagen with the human fibrinogen molecule being composed of three pairs of non-identical chains (α_2_β_2_γ_2_). As carboxyl groups exist at the C-terminus of each polypeptide chain, and within groups of aspartic acid (Asp,D) and glutamic acid (Glu,E), using the protein sequences of the three chains [60,61] it was calculated that the number of carboxyl groups per fibrinogen molecule totalled approximately 500. Analysis determined that whilst this 5:2:1 ratio for collagen equated to 1.14 g EDC and 0.276 g NHS per 1 g collagen, for every gram of fibrinogen the quantities were 1.31 g EDC and 0.333 g NHS.

Cross-linking reagents were dissolved in 95% ethanol and samples were soaked for 2 h within the well plate before thorough washing (5 × 5 min with deionised water). 

### 4.4. Analysis of Distribution of Collagen and Fibrinogen

**Figure 8:**
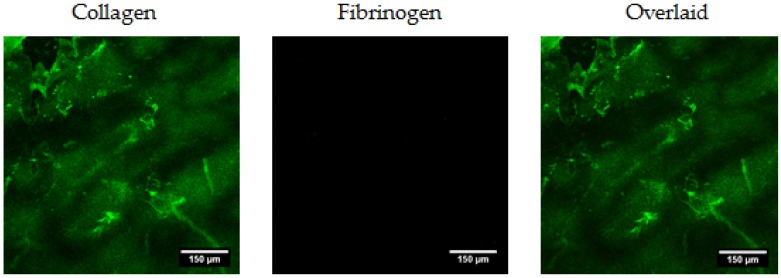
Specificity of the antibodies is demonstrated: 100% collagen films are imaged in the presence of both primary and secondary antibodies, and no fibrinogen-associated signal was detected.

A simultaneous incubation procedure was carried out with monoclonal antibodies for collagen and fibrinogen (all antibodies provided by AbCam, Cambridge, UK). All samples were initially incubated in 1% bovine serum albumin (BSA) in PBST (phosphate-buffered saline solution with 0.1% Tween 20) before a one hour incubation with primary antibodies at room temperature (anti-collagen I rabbit and anti-fibrinogen goat). After 3 × 5 min static washes with PBS, samples were then incubated with secondary antibodies (Donkey Anti-rabbit Alexa Fluor 488 and Donkey Anti-goat Alexa Fluor 647), washed repeatedly, and samples were mounted for confocal imaging (Leica SP2). It was not possible to produce scaffolds or, indeed, films consisting of 100% fibrinogen, so specificity of the antibodies were not fully verified, however, analysis of 100% collagen films in the presence of all primary and secondary antibodies did not demonstrate any fibrinogen-associated signal (Figure 8). Distribution of collagen and fibrinogen within both films and scaffolds was then determined. 

### 4.5. Characterisation of Scaffold Pore Structure

Micro-CT (Skyscan 1172, Bruker MicroCT, Kontich, Belgium) was considered with the cylindrical samples scanned in their entirety with a pixel size of 3.0 μm, an operating voltage of 25kV, 0.2° step size, with frame averaging of 2° and 180° rotation. The resulting projections were processed into 3D datasets using a full cone beam Feldkamp reconstruction algorithm with NRecon software (version 1.6.10.2, Bruker, Kontich, Belgium). Three cubic regions of interest (ROIs) were defined through each scaffold (1 mm^3^) to allow investigation of pore homogeneity and for ease of processing. Thresholding of these regions of interest was carried out using the Otsu algorithm [62] using Bruker’s CTAn software (version 1.15.4, Bruker, Kontich, Belgium). Binary images were then cleaned up using a sweep de-speckle feature. Using the analytical capabilities of the CTAn software, overall porosity was determined, pore sizes calculated using a sphere fitting method, and surface area calculated using a ‘marching cubes’ method [63].

There are a number of measures which may describe the interconnectivity of a scaffold structure; the percolation diameter is a scale-invariant parameter and can describe the characteristics of the transport route encountered by an infiltrating cell [42]. This percolation diameter is the diameter of the largest sphere able to penetrate through an infinitely large scaffold. For percolation analysis three ROIs of dimension 1 mm × 1 mm × 1 mm were modified to allow penetration only from the top and bottom *x*-*y* planes. The analysis was carried out using a method described by Ashworth et al. [42]. Briefly, CTAn’s shrink wrap feature was used to identify the volume accessible to a virtual object. By increasing the diameter of this object ‘*d*’, the corresponding length of accessible pore volume ‘l’ in the *z* direction could be measured and this data plotted using the relationship from percolation theory in order to calculate the percolation diameter:

(1) $$L=L_{o}{\left(d-d_{c}\right)}^{-v}$$

where *v* is 0.88 for a 3D system [64].

Additionally, through-thickness sections of the scaffolds were produced using a scalpel, gold coated and imaged, using scanning electron microscopy (JEOL 5800, JEOL, Welwyn Garden City, UK).

### 4.6. Collagen-Fibrinogen Films

Collagen-fibrinogen films were produced with the same compositions as considered for the lyophilized structures (0%, 10%, 25%, and 50% fibrinogen with a total 1 wt % solids). For immunofluorescence these films were produced on circular glass coverslips with 106 μL suspension per sample. Films were additionally cast on the base of standard 48 well plates for cell culture with 76 μL of the suspension pipetted per well. All samples were air-dried. Cross-linking was carried out at the 30% cross-linking level for immunofluorescence samples and the 10% and 100% level for cell samples, before rigorous washing (5 × 5 min wash with D.I. H_2_O). Cross-linking in ethanol solution was sufficient sterilisation for short-term cell culture and film samples were handled aseptically after this point.

### 4.7. Cell Culture

A preliminary study of cell attachment and spreading was carried out using the C2C12 mouse myoblast cell line. Films produced on the base of 48 well plates with all fibrinogen additions and 100% and 10% cross-linking concentrations were considered with four replicates per sample type. In order for the specific integrin binding of cells, divalent cations, such as magnesium are necessary [51,65], thus, our cell adhesion was carried out in the presence of Mg^2+^. In line with previous studies [47] integrin-based and non-integrin-based cell binding were separated through adhesion assays run in the presence of EDTA, used to remove divalent ions by chelation. The C2C12 mouse myoblast cell line was maintained in a humidified incubator with 5% CO_2_ at 37 °C in Dulbecco’s modified Eagle’s medium (DMEM, Sigma-Aldrich) containing 10% (*v*/*v*) foetal bovine serum (Sigma-Aldrich) and 1% (*v*/*v*) streptomycin/penicillin (Sigma-Aldrich). Cells were prepared for cell analysis by detaching from the cell culture flasks with 0.05% (*w*/*v*) trypsin/0.02% (*w*/*v*) EDTA (Sigma-Aldrich) and re-suspending in serum free DMEM to a density of 2 × 10^5^ cells/mL.

#### 4.7.1. Cell Adhesion Analysis

Non-specific adsorption was blocked with 250 μL of 5% (*w*/*v*) bovine serum albumin (BSA) (Sigma Aldrich, Gillingham, UK) in phosphate-buffered saline (PBS) (Sigma Aldrich, Gillingham, UK) for 60 min, and then washed three times with 250 μL of PBS. Two-hundred microlitres of cells were added to each well in serum-free DMEM (Sigma Aldrich, Gillingham, UK) containing either 5 mM MgCl_2_ or 5 mM ethylenediaminetetraacetic acid (EDTA) (Sigma Aldrich, Gillingham, UK). After incubation at 37 °C/5% CO_2_ for 45 min, loosely-bound cells were removed with 3 × 250 μL PBS washes. Bound cells were lysed with 100 μL of 2% (*v*/*v*) Triton X-100 for 4 h at room temperature then detected by adding 100 μL of lactate dehydrogenase (LDH) substrate (Sigma-Aldrich, Gillingham, UK) prepared according to the manufacturer’s specifications. After 30 min of incubation at room temperature the absorbance at 490 nm was measured using a BMG LABTECH SPECTROstar Nano plate reader (BMG LABTECH Ltd, Aylesbury, UK). Values are presented as means of quadruplicate measurements ± standard deviations.

#### 4.7.2. Cell Spreading Analysis 

Films were BSA-blocked as for attachment analysis. Near confluent cell monolayers were washed with serum free DMEM then incubated in serum free DMEM containing 5 μM CellTracker Green CMFDA (5-chloromethylfluorescein diacetate)(Molecular Probes, Eugene, OR, USA) for 30 min at 37 °C/5% CO_2_. The cells were washed with serum free DMEM then detached from the flask with 0.05% (*w*/*v*) trypsin/0.02% (*w*/*v*) EDTA (Sigma-Aldrich, Gillingham, UK) and re-suspending in serum free DMEM to a density of 2 × 10^5^ cells/ml. Two-hundred microlitres of cells were added to each well in serum-free DMEM for 120 min at 37 °C/5% CO_2_. Subsequently the cells were fixed by the addition of glutaraldehyde (Sigma-Aldrich, Gillingham, UK) to the cell medium to achieve a final concentration of 5% (*w*/*v*) for 20 min at room temperature. The wells were extensively washed with PBS, the wells filled with PBS, then a glass slide layered across the well plate. Cell images were taken on a Zeiss Axio Observer Z1 phase contrast microscope fitted with a Zeiss Axiocam 503 mono camera using a 10× objective lens (Carl Zeiss Ltd, Cambridge, UK). Cell spreading was quantified by scoring a cell as 'spread if it was phase-dark with cellular projections and had a flattened morphology. Cells were scored as non-spread if rounded and phase-bright with no cellular projections, as detailed in [66]. The percentage cell spreading was calculated by dividing the number of spread cells by the total number of cells present. Values are means of quadruplicate measurements ± standard deviation

### 4.8. Statistical Analysis

The normality of cell spreading data was tested using the Shapiro-Wilks test of normality. Statistical significance was determined through a one-way ANOVA, followed by Tukey’s HSD. Significance was defined as *p* < 0.05.

## 5. Conclusions

Novel lyophilized collagen-fibrinogen scaffolds have been produced across a range of fibrinogen contents and cross-linking concentrations. Applying, for the first time, immunofluorescence to the characterization of produced scaffolds, distributions of the two constituents were shown to be uniform at up to 50% fibrinogen and despite some variations in mean pore size, the interconnected nature of the scaffolds was broadly independent of composition and cross-linking. Using films as a model of the strut surfaces of scaffolds, the work has demonstrated a significant biological advantage to the addition of fibrinogen within collagen structures, particularly at levels of cross-linking where specific binding to collagen has previously been shown to be ablated.

## Figures and Tables

**Table 1:** Summary of the samples produced for analysis where: μCT—micro-computed tomography, sIF—scaffold immunofluorescence, fIF—film immunofluorescence, CA—cellular adhesion, and CS—cell spreading. All samples were produced in triplicate (although detailed analysis was carried out only on single representative samples in the case of micro-CT and immunofluorescence) except films for cellular analysis where all tests were carried out in quadruplicate.

Fibrinogen Content	Cross-Linking Concentration				
	1%	10%	30%	50%	100%
0%	μCT, sIF	μCT, sIF, CA	μCT, sIF, fIF	μCT, sIF	μCT, sIF, CA, CS
10%	μCT, sIF	μCT, sIF, CA	μCT, sIF, fIF	μCT, sIF	μCT, sIF, CA, CS
25%	μCT, sIF	μCT, sIF, CA	μCT, sIF, fIF	μCT, sIF	μCT, sIF, CA, CS
50%	μCT, sIF	μCT, sIF, CA	μCT, sIF, fIF	μCT, sIF	μCT, sIF, CA, CS
